# Tetramethylpyrazine Improves Cognitive Impairment and Modifies the Hippocampal Proteome in Two Mouse Models of Alzheimer's Disease

**DOI:** 10.3389/fcell.2021.632843

**Published:** 2021-03-15

**Authors:** Xianfeng Huang, Jinyao Yang, Xi Huang, Zaijun Zhang, Jianjun Liu, Liangyu Zou, Xifei Yang

**Affiliations:** ^1^School of Pharmacy and School of Medicine, Changzhou University, Changzhou, China; ^2^Key Laboratory of Modern Toxicology of Shenzhen, Shenzhen Medical Key Subject of Modern Toxicology, Shenzhen Center for Disease Control and Prevention, Shenzhen, China; ^3^Department of Neurology, Shenzhen People's Hospital (First Affiliated Hospital of Southern University of Science and Technology), Second Clinical College, Jinan University, Shenzhen, China; ^4^Institute of New Drug Research and Guangzhou, Key Laboratory of Innovative Chemical Drug Research in Cardio-Cerebrovascular Diseases, Jinan University College of Pharmacy, Guangzhou, China

**Keywords:** tetramethylpyrazine, Alzheimer's disease, proteomics, mitochondria, OxPhoS

## Abstract

Alzheimer's disease (AD), one of the most common neurodegenerative diseases, has no effective treatment. We studied the potential effects of tetramethylpyrazine (TMP), an alkaloid in the rhizome of *Ligusticum chuanxiong* Hort. used in Traditional Chinese Medicine (*chuānxiong*) to treat ischemic stroke, on AD progression in two AD mouse models. Eight-month-old 3xTg-AD mice received TMP treatment (10 mg/kg/d) for 1 month, and 4-month-old APP/PS1-AD mice received TMP treatment (10 mg/kg/d) for 2 months. Behavioral tests, including step-down passive avoidance (SDA), new object recognition (NOR), Morris water maze (MWM), and Contextual fear conditioning test showed that TMP significantly improved the learning and memory of the two AD-transgenic mice. In addition, TMP reduced beta-amyloid (Aß) levels and tau phosphorylation (p-tau). Venny map pointed out that 116 proteins were commonly changed in 3xTg mice vs. wild type (WT) mice and TMP-treated mice vs. -untreated mice. The same 130 proteins were commonly changed in APP/PS1 mice vs. WT mice and TMP-treated mice vs. -untreated mice. The functions of the common proteins modified by TMP in the two models were mainly involved in mitochondrial, synaptic, cytoskeleton, ATP binding, and GTP binding. Mitochondrial omics analysis revealed 21 and 20 differentially expressed mitochondrial proteins modified by TMP in 3xTg-AD mice and APP/PS1 mice, respectively. These differential proteins were located in the mitochondrial inner membrane, mitochondrial outer membrane, mitochondrial gap, and mitochondrial matrix, and the function of some proteins is closely related to oxidative phosphorylation (OXPHOS). Western-blot analysis confirmed that TMP changed the expression of OXPHOS complex proteins (sdhb, ndufa10, uqcrfs1, cox5b, atp5a) in the hippocampus of the two AD mice. Taken together, we demonstrated that TMP treatment changed the hippocampal proteome, reduced AD pathology, and reduced cognitive impairment in the two AD models. The changes might be associated with modification of the mitochondrial protein profile by TMP. The results of the study suggest that TMP can improve the symptoms of AD.

## Introduction

Alzheimer's disease (AD), a common form of neurodegenerative dementia, has a huge impact on the health system (Hyman et al., 2012; Oboudiyat et al., 2013). The amount of patients with AD is increasing dramatically in aging populations worldwide, such that identification of effective therapeutics is a top priority for society (Foloppe et al., 2018).

Alzheimer's disease (AD) is featured by prominent neuropathological changes, including β-amyloid (Aβ) plaques and neurofibrillary tangles (NFT) formed from hyperphosphorylated tau (p-tau). The pathogenesis of AD has suggested various hypthotheses including roles for inflammation, cholinergic function, Aβ deposition, tau hyperphosphorylation, and mitochondrial dysfunction (Scholtzova et al., 2008). As for AD treatment, prior to 2019, the US Food and Drug Administration approved only six drugs market use (Briggs et al., 2016). Other more effective medicines are urgently needed to address the growing AD patient load.

We studied the potential effects of tetramethylpyrazine (TMP), an alkaloid in the rhizome of *Ligusticum chuanxiong* Hort, used in Traditional Chinese Medicine (chuānxiong) to treat ischemic stroke, on AD progression in two AD mouse models.

Tetramethylpyrazine (TMP), a type of calcium antagonist, is an alkaloid extracted from the rhizome of *Ligusticum chuanxiong* Hort, which has been widely used in Chinese Traditional Medicine. According to reports in the literature, TMP has shown a strong neuroprotective effect in brain ischemia models (Chang et al., 2007). In the rat model of Parkinson's disease (PD) induced by systemic treatment with methylphenyltetrahydrodpyridine, TMP has a neuroprotective effect on dopaminergic neurons (Lu et al., 2014), and TMP reduces the severity of rotenone-induced PD-like disease in rats (Michel et al., 2017). Additionally, TMP reversed streptozotocin-induced memory impairment by inhibiting glycogen synthase kinase-3β (GSK-3β) (Lu et al., 2017). Taken in concert, these data suggest that TMP may have a potential therapeutic effect via neuroprotection. We therefore studied the potential neuroprotective effect of TMP on AD and its molecular mechanism through the use of two validated AD mouse models.

## Materials and Methods

### Animals and Treatment Protocol

Animals used included: (a) triple transgenic AD mice (3xTg-AD) (B6; 129-Psen1^tm1Mpm^ Tg [APPSwe, tauP301L] 1Lfa/Mmjax) and wild-type (WT) mice (B6129SF2/J) and (b) APPswe/PSEN 1dE9 (APP/PS1) mice and matched wild-type (WT) animals (APP/PS1-negative mice). Mice were obtained from the Jackson Laboratory in the United States, delivered by air to Shenzhen, and bred locally at the Shenzhen Center for Disease Control. Animal housing, breeding, and experimental studies used standards employed for breeding laboratory animals.

**Figure 1:**
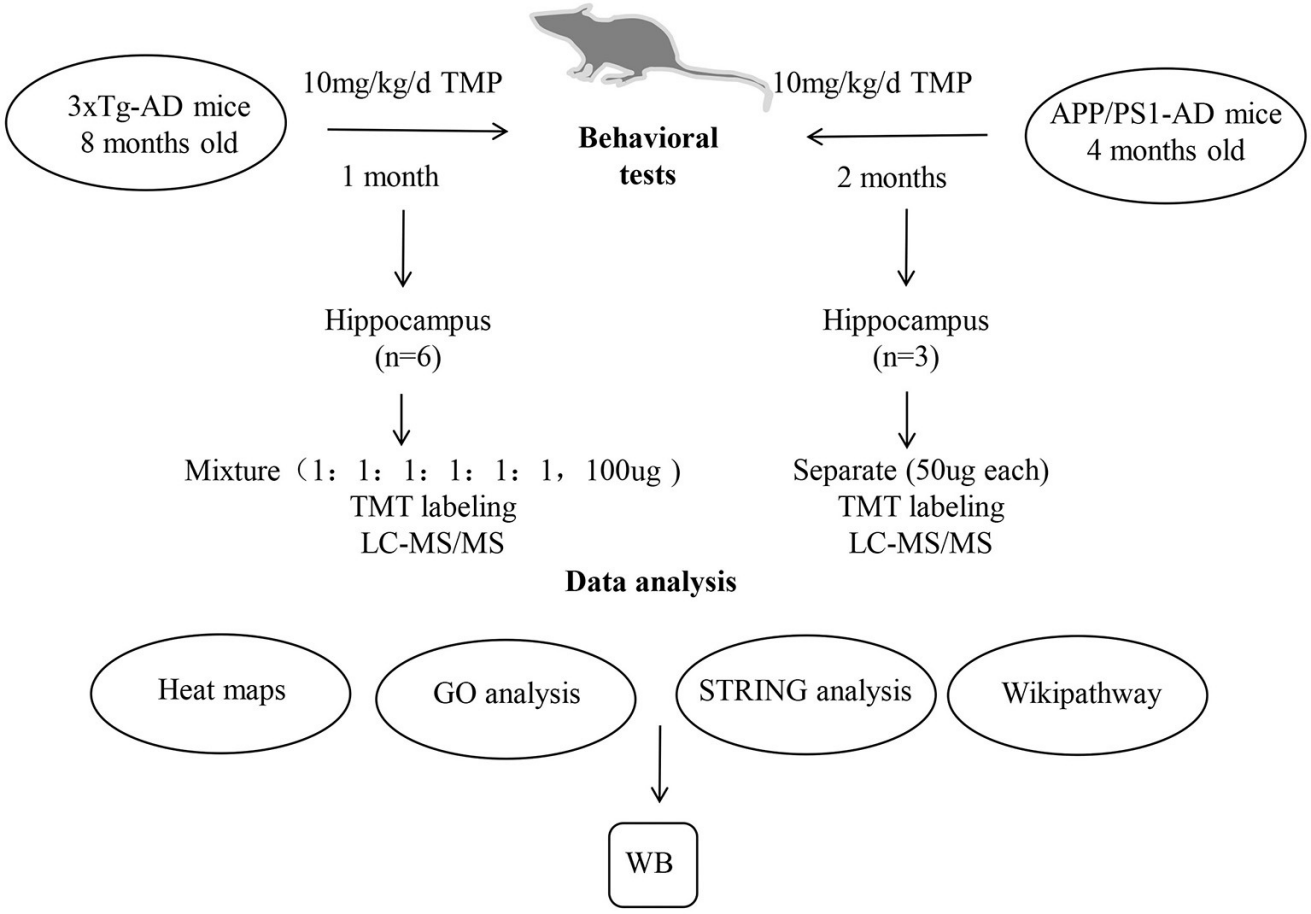
A schematic diagram of the experimental design. 3xTg-AD and APP/PS1-AD female mice were treated by gavage with TMP (10 mg/kg/d) in saline or an equal volume of saline. After the behavioral assessment, the brain removed and the hippocampus isolated for proteomic analysis. Protein samples from the brains of 6 mice per group (WT mice, 3xTg mice, and TMP-treated mice) were collected. Protein samples from the brains of 3 mice of each group (WT mice, APP/PS1 mice, and TMP-treated mice) were collected. The peptides were further graded with 6-plex TMT and HPRP and then analyzed by LC-MS/MS. Bioinformatics analysis (heat map, GO, String, and Wikipathway) was performed with David 6.8 and Cytoscape software. WB, Western blots.

Eight-month-old 3xTg-AD mice were treated by gavage with 10 mg/kg/d TMP (10 mg/kg/d) in saline for 1 month. Eight-month-old WT mice and untreated 3xTg-AD mice were given normal saline by the same route. Additionally, 4-month-old APP/PS1-positive mice were treated by gavage with 10 mg/kg/d TMP (TMP+10 mg/kg/d) in saline for 2 months, and 4-month-old negative mice and untreated positive mice were given normal saline (Figure 1). The experiments were performed in accordance with the National Institutes of Health Guide for the Care and Use of Laboratory Animals (NIH Publication No. 8023, Revised 1978) and approved by the Ethics Committee of Shenzhen Center for Disease Control and Prevention. Every effort was made to reduce the suffering of the animals and the number of mice.

### Behavioral Tests

#### Cognition Test

We performed a new object recognition (NOR) test using a previously published method (Wang et al., 2019). On the first day, mice were conditioned to move freely in an empty plastic box for 5 min. After 24 h of training, the mice were placed back in the same box with two objects of the same size and shape and allowed to explore freely for 5 min. After 1 h, one of the objects was replaced with a new object of the same size but different shape, and the mice were allowed to explore freely for another 5 min. Simultaneous video and tracking documented the detection time of each object. Detection was defined as the mouse facing the object, sniffing or touching with the nose, and the distance was recorded from the nose to the object ≤ 2 cm. The calculation method was: (time to explore new objects)/(time to explore new objects + time to explore old objects) ^*^ 100%.

#### Step-Down Passive Avoidance Test

We used step-down passive avoidance (SDA) test to assess aversive learning and memory in the models (Zhou et al., 2019a). The device consisted of five glass rooms with length, width and height of 15 × 15 × 46 cm^3^. The floors were spaced 1 cm apart and a platform with a diameter of 4.5 cm was placed in the middle. On day one, the mouse was placed into the electrical stimulation box and electrical stimulation administered to the animal. The response time for the animal to jump onto platform and number of platforms used was recorded (error number). After 24 h, the mouse was directly placed on the platform and the time (latency) and error times of the mouse jumping off the platform for the first time were recorded.

#### Morris Water Maze Test

We used the Morris water maze (MWM) test according to a previously published method to evaluate the spatial learning and memory abilities of mice (Zhou et al., 2019b). The water maze consists of a circular pool and a white escape platform; the pool is filled with opaque water and has a white escape platform with a diameter of 10 cm under the water surface. If the platform was found by the animal within 1 min of water immersion, the time to platform was recorded; if the platform was not found within 1 min, the animal was helped to find the platform and allowed to stay there for 15 s. The training lasted for 5 days, starting from the four starting positions, and performed four times a day, with an interval of 15 s. Animal performance was tested on the seventh day after training. The times of crossing the platform area, time on the platform, activity track, activity time, and distance of target quadrant were all recorded.

#### Contextual Fear Conditioning Test

Previously reported methods were used to conduct the conditional fear experiment (Bolton et al., 2012; Zhou et al., 2012). In short, the fear regulator consists of a conditioning chamber. The sound chamber is closed by silencers and the noise is shielded by exhaust fans. The infrared digital camera is placed at the top of the enclosure, about 50 cm from the chamber. On the first day of training, the mice explored freely for 2 min without any stimulation. The loudspeaker (4 Hz, 80 dB) was started and stopped after 30 s. Electrical stimulation (0.5 mA) was started 2.5 s later and ended after 1 s. The mice were exposed to no stimulation for 2 min and then the entire procedure was repeated three times. The chamber was cleaned with 70% alcohol 24 h after training. The mice were exposed to the changed environment for 11 min. The training included 2 min of free exploration, 60 s of speaker (4 Hz, 80 dB), and three repetitions. Fourty-eight hours after the training in the original training room. The mice were left in the same room and allow to explore for 6 min without any electrical or sound stimulation.

### Protein Extraction and Digestion

Hippocampal protein samples were prepared (Xu et al., 2018). Hippocampal tissue of mice was dispersed in 8 M urea phosphate buffer solution by ultrasound and then centrifuged. The hippocampus of 3xTg mice (6 mice in each group) was mixed into 100 μg protein according to (1:1:1:1:1:1). The hippocampal proteins of APP/PS1 mice (three in each group) were similarly taken in 100 μg protein. Protein samples were incubated with 10 mM 1,4-Dithiothreitol (DTT) at 55°C for 30 min and then with 25 mM diamine iodoacetate (IAA) for 1 h at room temperature. Samples were then digested with trypsin at 37°C, adjusted to pH 1–2 by adding 1% trifluoroarboxylic acid (TFA), and centrifuged at 12,000 g for 20 min to collect the supernatant. Inverse phase column chromatography (HLB, Waters OASIS, USA) was used for desalting. After desalination, samples were dried and dissolved in a buffer of triethylamine bicarbonate (TEAB, 200 mM, pH 8.5).

### Tandem Mass Tag (TMT) Labeling

The samples were labeled with TMT reagent at room temperature, 5 μl 5% hydroxylamine added, and the reaction terminated after incubation. The samples were labeled with different TMT Tags: TMT-126 for samples from WT mice; TMT-127, 3xTg mice; TMT-128, TMP-treated mice, TMT-129, APP/PS1 wild mice; TMT-130, APP/PS1-positive mice, TMT-131, TMP-treated mice. The total peptides labeled in each group were mixed, desalted, dried, and fractionated.

### LC-MS/MS Analysis and Database Searching

The labeled peptides were then loaded onto an Xbridge BEH300 C18 column (Waters, USA) and the peptide samples were separated into 15 fractions using an UltiMate 3000 UHPLC (Thermo Fisher Scientific, USA) (He et al., 2019). Then, fractions were dried and used for LC-MS/MS analyses. LC-MS/MS analyses were used previously method (Chen et al., 2019). An analytical capillary column was used to separate peptides and filled with C18 silicone resin (Varian, Lexington, Massachusetts, USA). Data were interpreted using the UniProt muscle database (released in October 2018), and the original mass spectra were searched using Proteome Finder 2.1 software.

Mass spectrometry proteomics data were deposited in the ProteomeXchange Consortium via the PRIDE partner repository with the dataset identifier PXD022862 and PXD022840.

### Bioinformatics Analysis

We used Venny 2.1 to analyze differentially expressed proteins. Functional enrichment analysis of biological processes (BP) and molecular function (MF) was performed using DAVID Bioinformatics Resources 6.8. STRING database version 10.5 was used to analyze protein–protein interaction networks.

### Western-Blot Analysis

Hippocampal tissues were sonicated with RIPA lysate (Thermo Science, New Jersey, USA) containing 1X protease and phosphatase inhibitors. The protein concentration was quantified using the Thermo Fisher Scientific BCA protein analysis kit and then mixed with the loading buffer, heated at 95°C for 8 min; the protein was isolated by 10% SDS-PAGE and transferred to PVDF membrane, and then the 5% skimmed milk was dissolved in TBST buffer and blocked for 2 h. The blocked membrane was incubated overnight with the primary antibody in an ice box, including β-actin (1:3,000), α-tubulin (1:3,000), NDUFA10 (1:1,000), SDHB (1:1,000), UQCRFS1 (1:1,500), COX5B (1:1,000), ATP5A (1:1,000), APP (1:1,000), BACE1 (1:1,000), PS1 (1:1,000), ADAM10 (1:1,000), IDE (1:1,000), PS396 (1:1,000), PS202 (1:1,000) in TBST. The membrane was washed three times in TBST and then incubated with the secondary antibody, diluted (1:3,000) in TBST for 50 min, and finally the ECL kit (Thermo Science, New Jersey, USA) was used to run the Western blot. The membrane was exposed, and ImageJ software used for quantitative analysis.

### Dot Blot Analysis

Mouse cortical tissue was sonicated with RIPA lysate. Total protein concentration used BCA protein assay kit and the total protein was diluted to the same concentration and applied to the NC film. The NC film was placed at room temperature for 3 h, transferred to 5% skimmed milk dissolved in TBST buffer and blocked for 2 h. The blocked membrane was incubated overnight with the 6E10 (1:1,000) antibody in an ice box. The membranes were washed three times in TBST and incubated with the secondary antibody diluted 1:3,000 in TBST for 50 min. The membrane was exposed, and ImageJ software used for quantitative analysis.

### ATP Levels

Cortical tissues of APP/PS1 mice were used to detect ATP levels in the brains of APP/PS1 mice. Briefly, to extract and determine protein concentration, samples (40 μl) were mixed with ATP assay solution (100 μl) and incubated for 3 min at room temperature. ATP levels were read using a microtiter plate with photometric capabilities.

### MDA Levels

Cortical tissues of APP/PS1 mice were used to detect MDA levels in the brains of APP/PS1 mice. Briefly, freshly extracted samples were subjected to protein concentration determination, and then the samples (100 μl) were incubated with MDA working solution (200 μl) at 100°C for 15 min and centrifuged at 1,000 g for 10 min. At the end, add 200 μl of supernatant to a 96-well plate and measure the absorbance at 532 nm. Lipid peroxidation levels were calculated as nmol/mg protein.

### Statistical Analysis

All the data were statistically processed using GraphPad Prism 8.0 software and the data expressed as the mean ± SEM. The data were tested by One-way ANOVA. *p* < 0.05 was set as statistically significant.

## Results

### TMP Improves Cognitive Function in 3xTg-AD Mice

**Figure 2:**
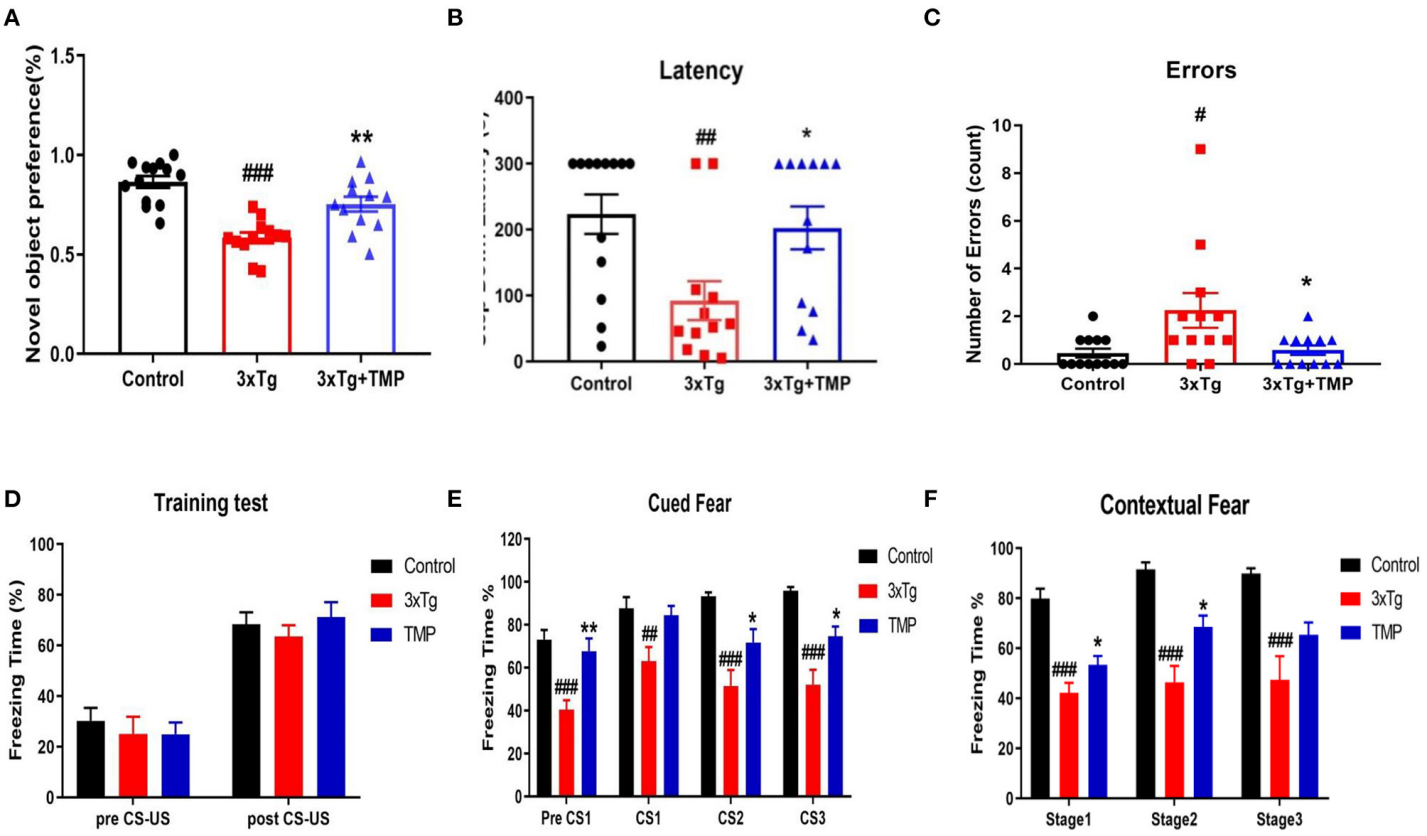
TMP improved cognitive function of 3xTg-AD mice. Differences in **(A)** the exploration time of a novel object in the novel object recognition test and **(B,C)** the latency and number of errors in the step-down passive avoidance test **(B,C)**. Mean proportion time freezing on the training day **(D)**. Mean proportion time freezing 24 h after training in the altered context **(E)**. Mean proportion time freezing 48 h after training **(F)**. *N* = 12–13 per group. Data are presented as mean ± SEM, **p < 0.01, *p < 0.05 vs. 3xTg-AD mice. ^*###*^*p* < 0.001, ^*##*^*p* < 0.01, ^#^*p* < 0.05 vs. WT mice.

The NOR test showed that the exploration time of new objects by 3xTg mice was shorter than for WT mice. The exploration time of new objects increased after TMP treatment (Figure 2A). Compared with WT mice, the step-down latency decreased, and the number of errors increased in 3xTg mice, TMP treatment significantly increased the step-down latency and reduced the number of errors (Figures 2B,C). Result of the contextual fear conditioning test showed no difference in the proportion of freezing time in the first day of training among all groups (Figure 2D). After 24 h training, in the clue fear test, 3xTg-AD mice showed significantly shorter freezing time relative to controls, and the freezing time was recovered after TMP treatment (Figure 2E). After 48 h training, in the contextual fear test, 3xTg-AD mice showed significant shorter freezing time than controls, and the freezing time was recovered after TMP treatment (Figure 2F). These data suggested that TMP reduced the spatial memory impairment of 3xTg-AD mice.

### TMP Improves Cognitive Function in APP/PS1-AD Mice

**Figure 3:**
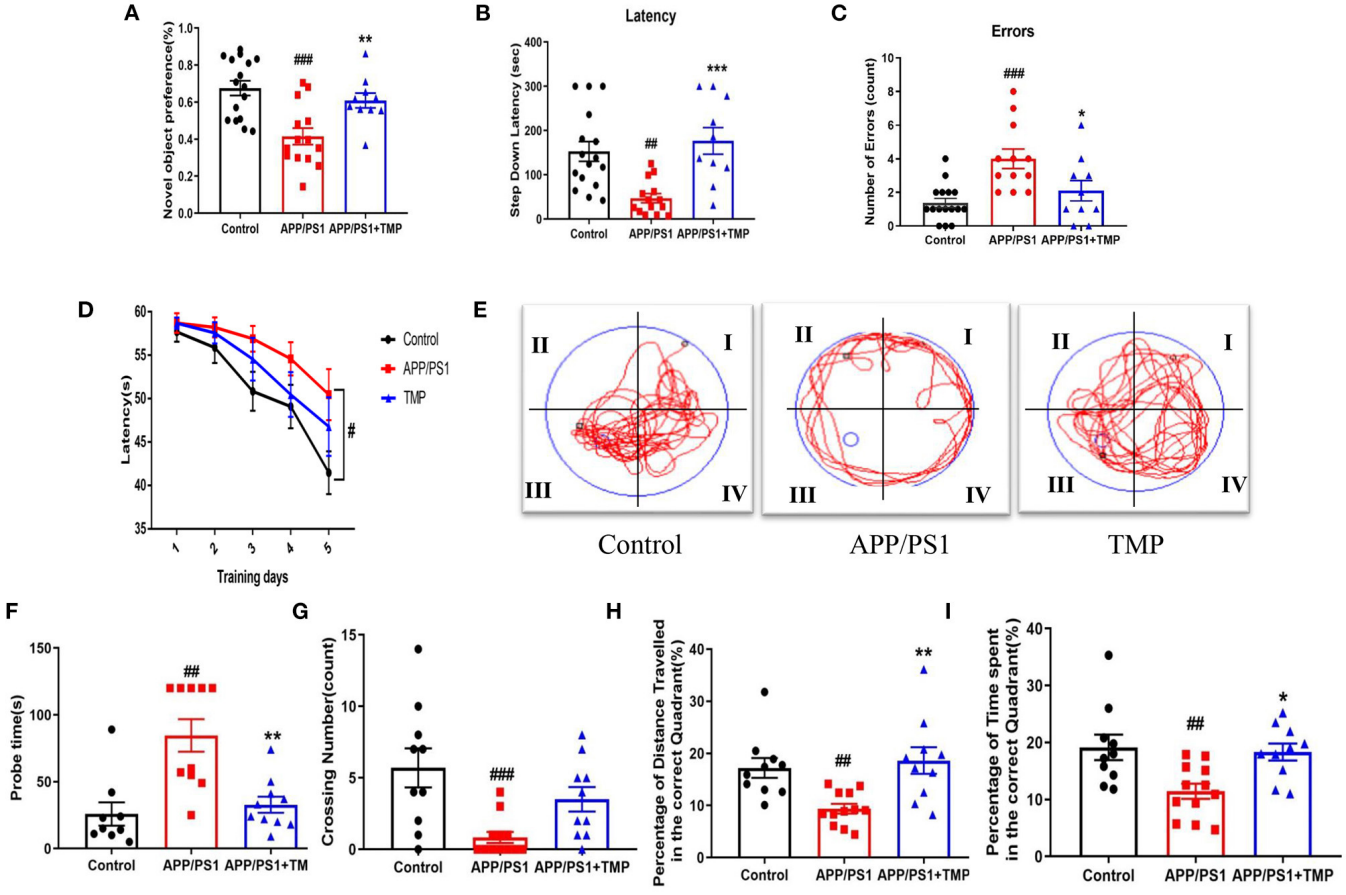
TMP improved cognitive function in APP/PS1-AD mice. Group differences in: **(A)** the exploration time percentage of novel objects in the novel object recognition test; **(B,C)** the latency waiting time and the number of errors in the downgrade passive avoidance test, and **(D)** the escape latency in the Morris water maze training test. Group differences in representative trajectories of motion patterns **(E)**, detection time **(F)**, the number of crossover movements **(G)**, the percentage of time spent in the platform quadrant **(H)**, and the percentage of distance moved in the platform quadrant **(I)** in probe trials of the Morris water maze test (*n* = 10–16 per group). The data are expressed as mean ± SEM. ***p < 0.001, **p < 0.01, *p < 0.05 vs. APP/PS1 mice; ^*###*^*p* < 0.001, ^*##*^*p* < 0.01 vs. Control mice.

The NOR test showed that the exploration time of new objects by APP/PS1 mice was shorter than that for the WT mice, and the exploration time for new objects was increased after TMP treatment (Figure 3A). The SDA test showed that, in APP/PS1 mice, the step-down latency was shorter than that of the WT mice. TMP treatment mice increased the step-down latency (Figure 3B). The error number in APP/PS1 mice was higher than that of the WT mice, and TMP reduced the error number (Figure 3C). The MWM test showed that during the continuous 5 days of training, the escape latency of the APP/PS1 mice was prolonged relative to that for the WT mice, and the escape latency was significantly decreased after TMP treatment (Figure 3D). The navigation path showed that the performance of the APP/PS1 mice was worse than that of the WT mice, and TMP treatment improved APP/PS1 performance (Figure 3E). In the probe test, compared with the WT mice, the probe time of APP/PS1 mice was longer and the time after TMP treatment was shortened (Figure 3F). Compared with the WT mice, the number of crossing in APP/PS1 mice was significantly fewer, and the number of crossing increased after TMP administration (Figure 3G). Compared to WT mice, APP/PS1 mice had reduced percentage of time in the target quadrant and percentage of distance traveled in the target quadrant, and both were increased after TMP treatment (Figures 3H,I). These data suggested that TMP improved the spatial memory impairment of APP/PS1 mice.

### Hierarchical Heat Map and Clustering Analysis

**Figure 4:**
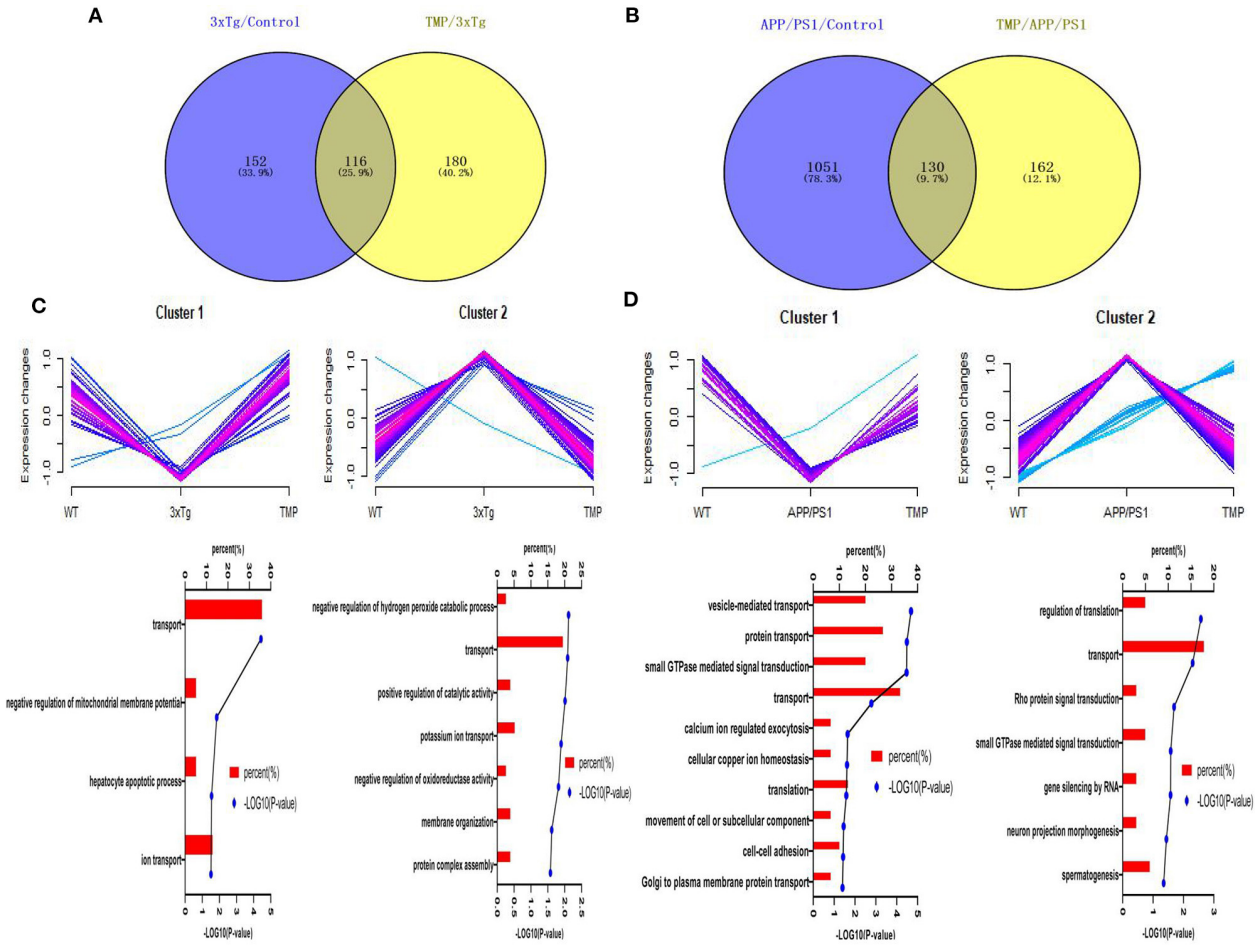
Venny analysis and cluster analysis of the differential proteins in the two mouse AD models. Venny maps of differential proteins in 3xTg-AD mice **(A)** and APP/PS1-AD mice **(B)**. Cluster analysis of differential proteins in 3xTg-AD mice **(C)** and APP/PS1-AD mice **(D)**.

**Figure 5:**
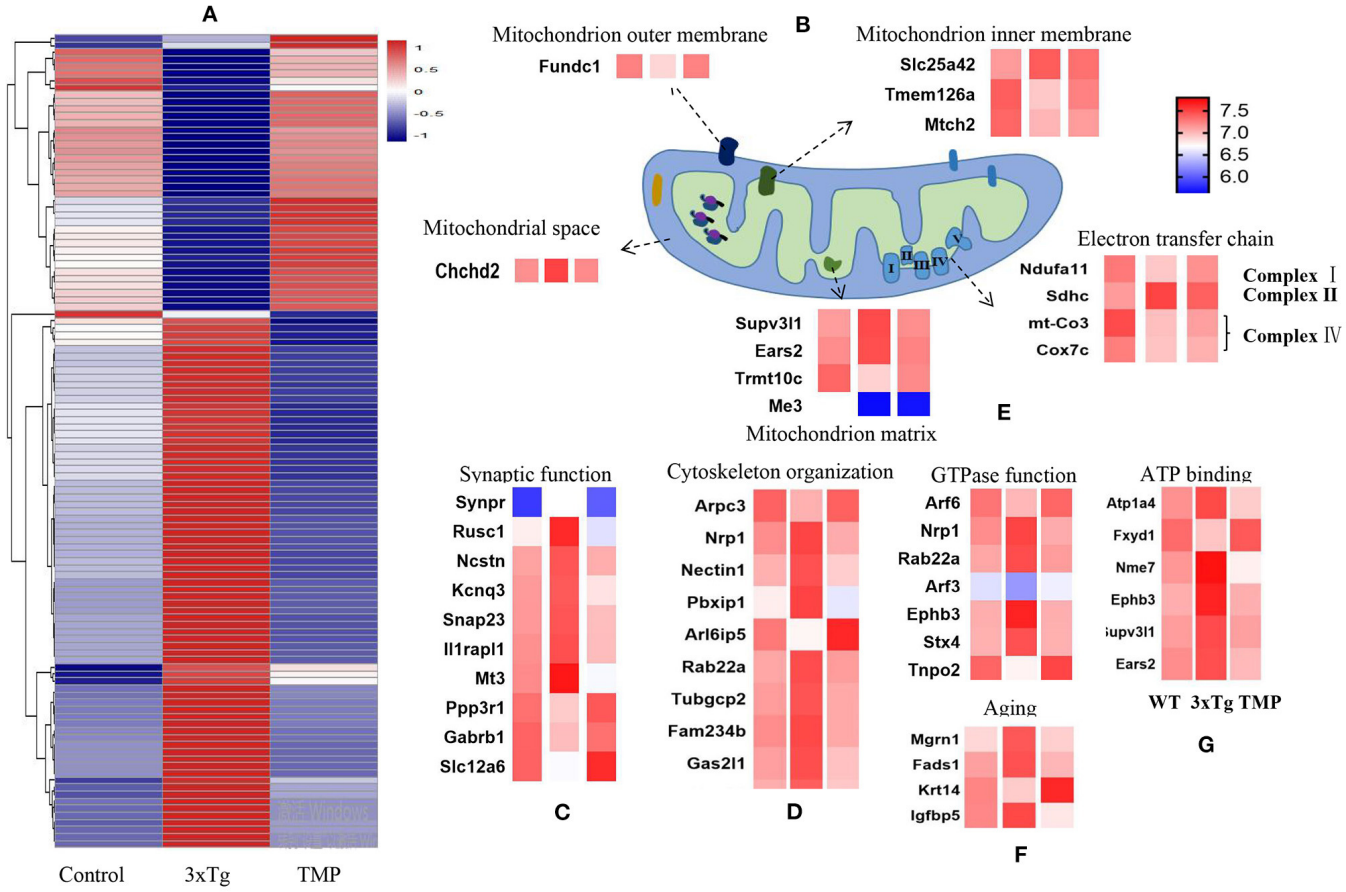
Heat map of differentially expressed proteins and mitochondrial protein projection. Classification maps of 116 differentially expressed proteins in 3xTg-AD mice **(A)**. Mitochondrial function, synaptic function **(C)**, cytoskeleton **(D)**, GTPase binding **(E)**, aging **(F)**, and ATP binding **(G)**. Mitochondrial proteins were specifically located in the mitochondrial inner membrane, mitochondrial outer membrane, mitochondrial matrix, mitochondrial gap, and electron transport chain **(B)**. Abundance value was calculated as log2 (abundance value).

**Figure 6:**
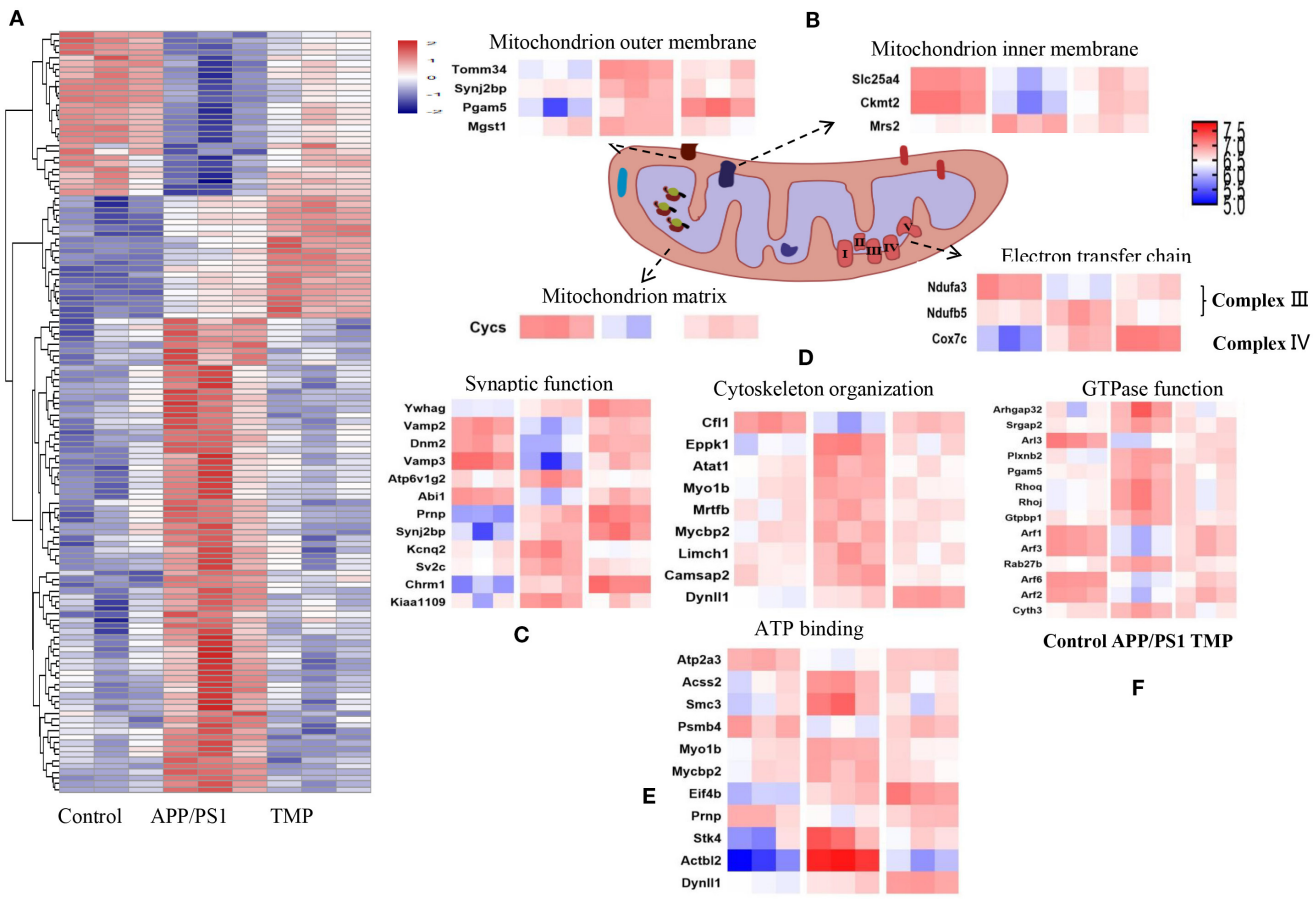
Differential protein heat map and mitochondrial protein profile. Classification maps of 130 differentially expressed proteins in APP/PS1-AD mice **(A)**. Mitochondrial function, synaptic function **(C)**, cytoskeleton organization **(D)**, ATP binding **(E)**, and GTP binding **(F)**. Mitochondrial proteins were specifically located in the mitochondrial inner membrane, mitochondrial outer membrane, mitochondrial matrix, mitochondrial gap and electron transport chain **(B)**. Abundance value was calculated as log2 (abundance value).

Hippocampal proteomics of 3xTg-AD mice identified a total of 5,390 proteins by one or more unique peptides. Venny analysis showed that, compared with the WT mice, 3xTg mice had 268 differentially expressed proteins, while TMP-treated 3xTg mice had 296 differentially expressed proteins compared with untreated 3xTg mice, of which 116 proteins were commonly changed (standard ratio ≥1.2 or ≤ 0.83) (Figure 4A). These 116 proteins were involved in mitochondrial function, ATP binding, synaptic function, cytoskeleton, GTP binding, and other functions (Figure 5). Hippocampal proteomics of APP/PS1 mice identified a total of 4,858 proteins by one or more unique peptides. Compared with the WT mice, there were 1,181 differentially expressed hippocampal proteins in APP/PS1 mice, while there were 282 proteins in the hippocampus of APP/PS1 mice treated with TMP vs. untreated APP/PS1 mice, of which 130 proteins were commonly changed (adjusted *P* < 0.05 and ratio ≥1.2 or ≤ 0.83) (Figure 4B). These 130 proteins were related to mitochondrial function, ATP binding, synaptic function, GTPase function, cytoskeleton, and so on (Figure 6). According to t cluster analyses for the two models, expression levels of most of the proteins in the TMP-treatment group returned to the level of the WT group (Figures 4C,D).

We further analyzed the BP of proteins classified by different cluster patterns. In 3xTg mice, Cluster 1 showed the proteins with an increasing trend of TMP administration, and the BP of these proteins included transport, negative regulation of mitochondrial membrane potential, hepatocyte apoptotic process, and ion transport. Cluster 2 mainly showed the proteins with a decreasing trend of TMP administration, the BP of these proteins included the negative regulation of hydrogen peroxide catabolic process, transport, positive regulation of catalytic activity of potassium ion transport, negative regulation of oxidoreductase activity, be organization, and protein complex assembly. In APP/PS1 mice, Cluster 1 showed the proteins with increasing TMP administration trend, and the BP of these proteins including vesicle-mediated transport, protein transport, small GTPase-mediated signal transduction, transport, calcium ion-regulated exocytosis, interfaces of cell and cell adhesion in cellular copper ion homeostasis, translation, movement of cell or subcellular component, and Golgi to plasma membrane protein transport. Cluster 2 mainly showed proteins with declining tendency of TMP administration; the BP of these proteins included regulation of translation, transport, Rho protein signal transduction, small GTPase-mediated signal transduction, gene silencing by RNA, neuron projection morphogenesis, and spermatogenesis.

We carried out sub-cellular localization analysis on the common changes of mitochondrial proteins. The mitochondrial proteins were projected to the inner mitochondrial membrane, outer mitochondrial membrane, mitochondrial matrix, mitochondrial space, and electron transport chain (Figures 5B, 6B). Compared with AD mice, TMP treatment changed the expression of most mitochondrial proteins.

### Enrichment Analysis of Differentially Expressed Proteins

**Figure 7:**
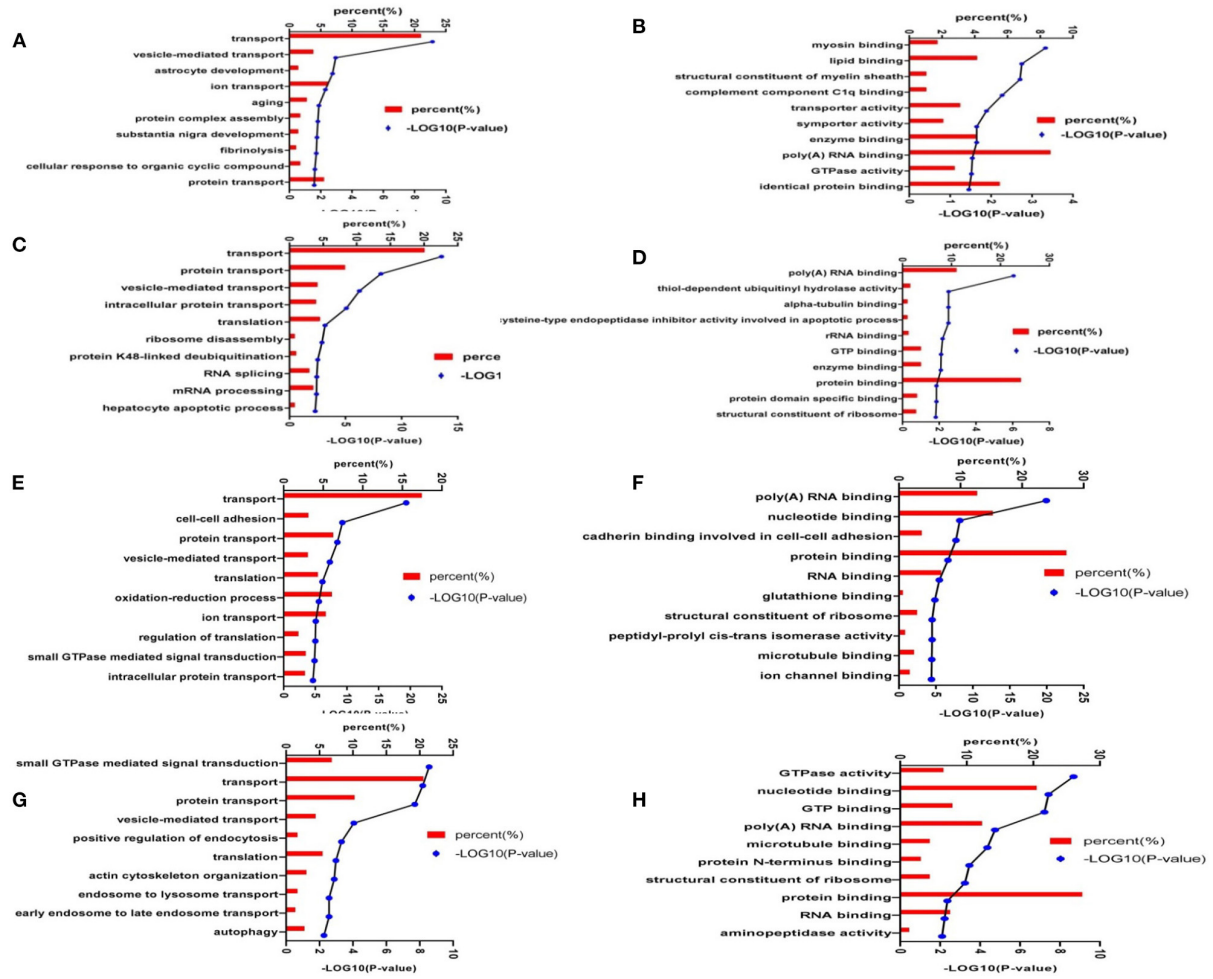
Enrichment analysis of the differentially expressed hippocampal proteins. The proteins were functionally annotated according to their biological processes, molecular functional terms, and listed by -log10(*p*-value). Biological processes of 3xTg mice vs. WT mice **(A)**; molecular functions of 3xTg mice vs. WT mice **(B)**; biological processes of TMP-treated mice vs. -untreated mice **(C)**; molecular functions of TMP-treated mice vs. -untreated mice **(D)**; biological processes of APP/PS1 mice vs. WT mice **(E)**; molecular functional of APP/PS1 mice vs. WT mice **(F)**; biological processes of TMP-treated mice vs. -untreated mice **(G)**, and molecular function of TMP-treated mice vs. -untreated mice **(H)**.

To better understand the biological function of differentially expressed proteins, in terms of BP and MFs, we identified the top 10 enriched groups. Gene ontology (GO) analysis of differential expressed proteins in 3xTg mice and WT mice, revealed BP involving transport, vesicle-mediated transport, astrocyte development, ion transport, aging, protein complex assembly, substantia nigra development, fibrinolysis, cellular response to organic cyclic compound, and protein transport (Figure 7A). Molecular functions included: protein binding, poly (A) RNA binding, myosin binding, lipid binding, structural constituent of myelin sheath, transporter activity, symporter activity, enzyme binding, GTPase activity, and identical protein binding (Figure 7B). For GO analysis of differential proteins in TMP-treated mice and 3xTg mice, the BP included: transporter, protein transport, vesicle-mediated transport, intracellular protein transport, translation, ribosome disassembly, protein K48-linked de-ubiquination, RNA splicing, mRNA processing, and hepatocyte apoptotic process (Figure 7C). Molecular functions included poly (A) RNA binding, thiol-dependent ubiquitin hydrolase activity, alpha-tubulin binding, rRNA binding, GTP binding, enzyme binding, protein binding, protein domain specific binding, structural constituent of ribosome (Figure 7D). For GO analysis of differential proteins in APP/PS1 mice and WT mice, the BP involved transport, cell–cell adhesion, vesicle-mediated transport, translation, oxidation-reduction process, ion transport, regulation of translation, intracellular protein transport, protein transport (Figure 7E). Molecular functions involved poly (A) RNA binding, nucleotide binding, protein binding, RNA binding, glutathione binding, microtubule binding, ion channel binding (Figure 7F). For GO analysis of differential proteins in TMP-treated mice and APP/PS1 mice, the BP involved transport, protein transport, small GTPase mediated signal transduction, translation, autophagy (Figure 7G). Molecular functions included GTPase activity, nucleotide binding, GTP binding, poly (A) RNA binding, microtubule binding, protein binding, RNA binding, aminopeptidase activity (Figure 7H).

### STRING Analysis

**Figure 8:**
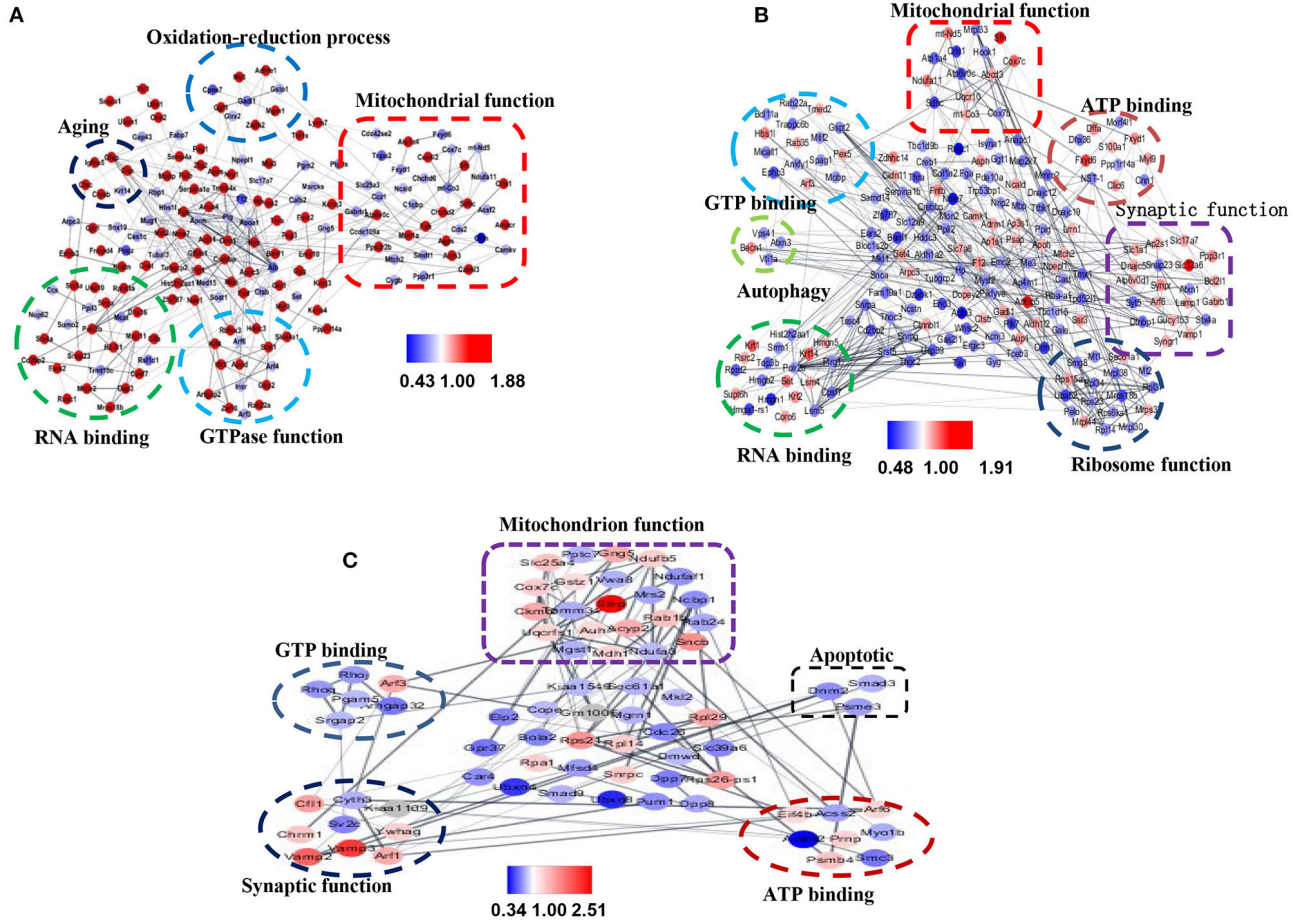
Protein–protein interaction (PPI) maps of significantly changed hippocampal proteins mapped by Cystoscape 3.7.1. PPI network of differentially expressed proteins in 3xTg mice vs. WT mice **(A)**. PPI network of differentially expressed proteins in TMP-treated mice vs. -untreated 3xTg mice **(B)**. PPI network of differentially expressed proteins in TMP-treated APP/PS1 mice vs. APP/PS1 mice **(C)**. Gray lines represent interactions between two proteins. Red nodes represent increased proteins, and blue nodes represent decreased proteins.

To evaluate the relationship between differentially expressed proteins (ratio ≥1.2 or ≤ 0.83), we used Cytoscape software to perform STRING analysis and to visualize protein–protein interaction networks. Most of the proteins were interlinked, many of which were involved in AD. The interactions of differentially expressed proteins between 3xTg and WT mice mainly included mitochondrial function, synaptic function, GTP binding, and aging (Figure 8A). The interaction of the differential proteins between TMP treatment 3xTg mice and 3xTg mice mainly included mitochondrial function, synaptic function, GTP binding, and autophagy (Figure 8B). The interaction of the differential proteins between TMP treatment and APP/PS1 mice mainly included mitochondrial function, synaptic function, GTP binding (Figure 8C).

### Comparison of Differential Expressed Proteins in Two AD Models After Administration

**Figure 9:**
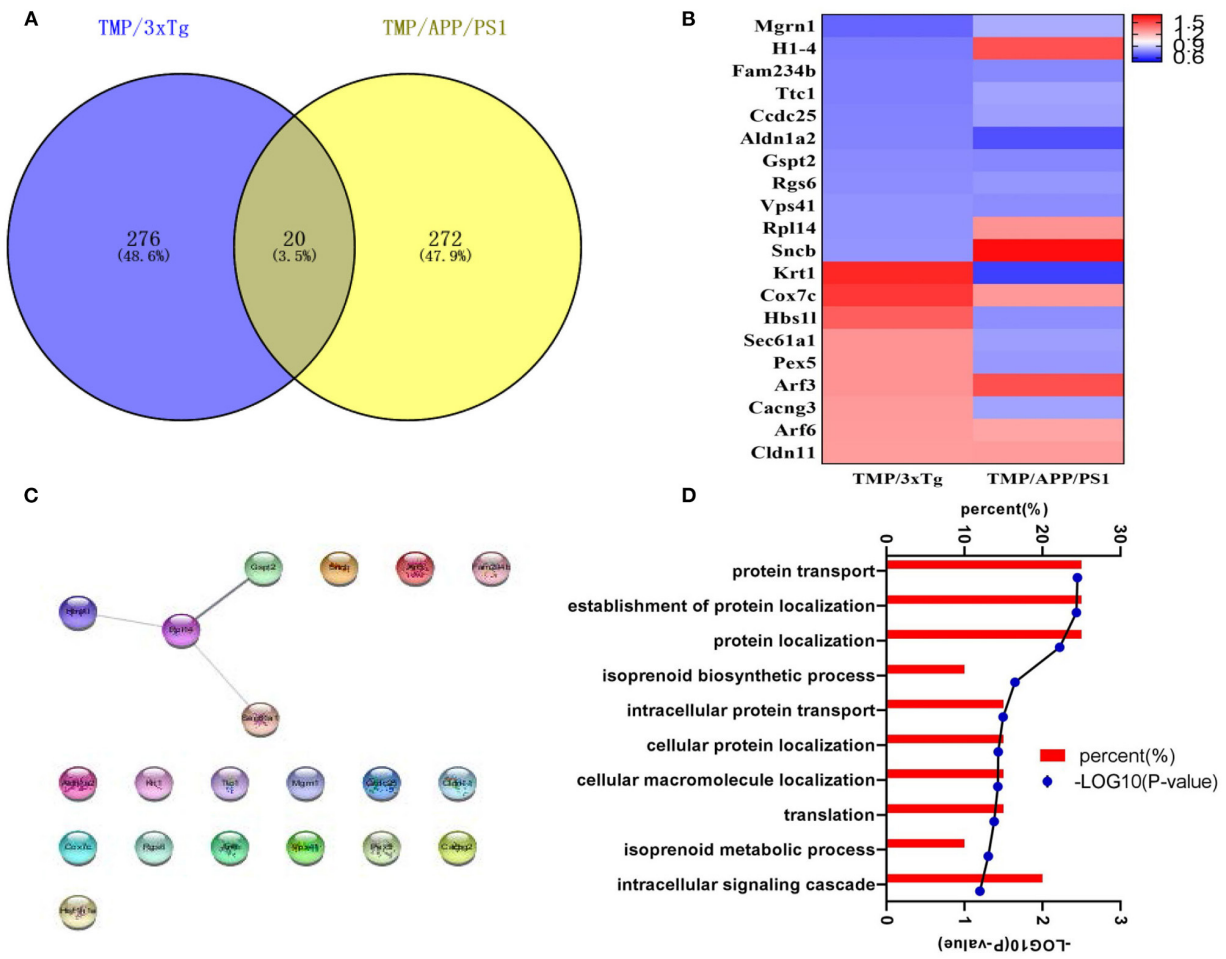
The analysis of differential proteins in 3xTg-AD and APP/PS1-AD with or without TMP treatment. **(A)** Venny analysis. **(B)** Heat map; the abundance value was calculated as log2 (abundance, value). **(C)** Biological processes. **(D)** The STING diagram showed the PPI network of 24 proteins. Among the 20 altered proteins, only four were found to interact.

We found that 20 proteins commonly changed after TMP administration in the two AD mouse models (Figure 9A). Among them, H1-4, Rpl14, Sncb, Krt1, Hbs1l, Sec61a1, Pex5, Cacng3 showed different trends, while the others all had the same increasing or decreasing trend (Figure 9B). The STING diagram showed the PPI network of 20 proteins, of which only 4 proteins interacted with each other (Figure 9C). The BP included protein transport, protein localization establishment, and protein localization (Figure 9D).

### TMP Affected the Activity of Electron Transport Chain Proteins

**Figure 10:**
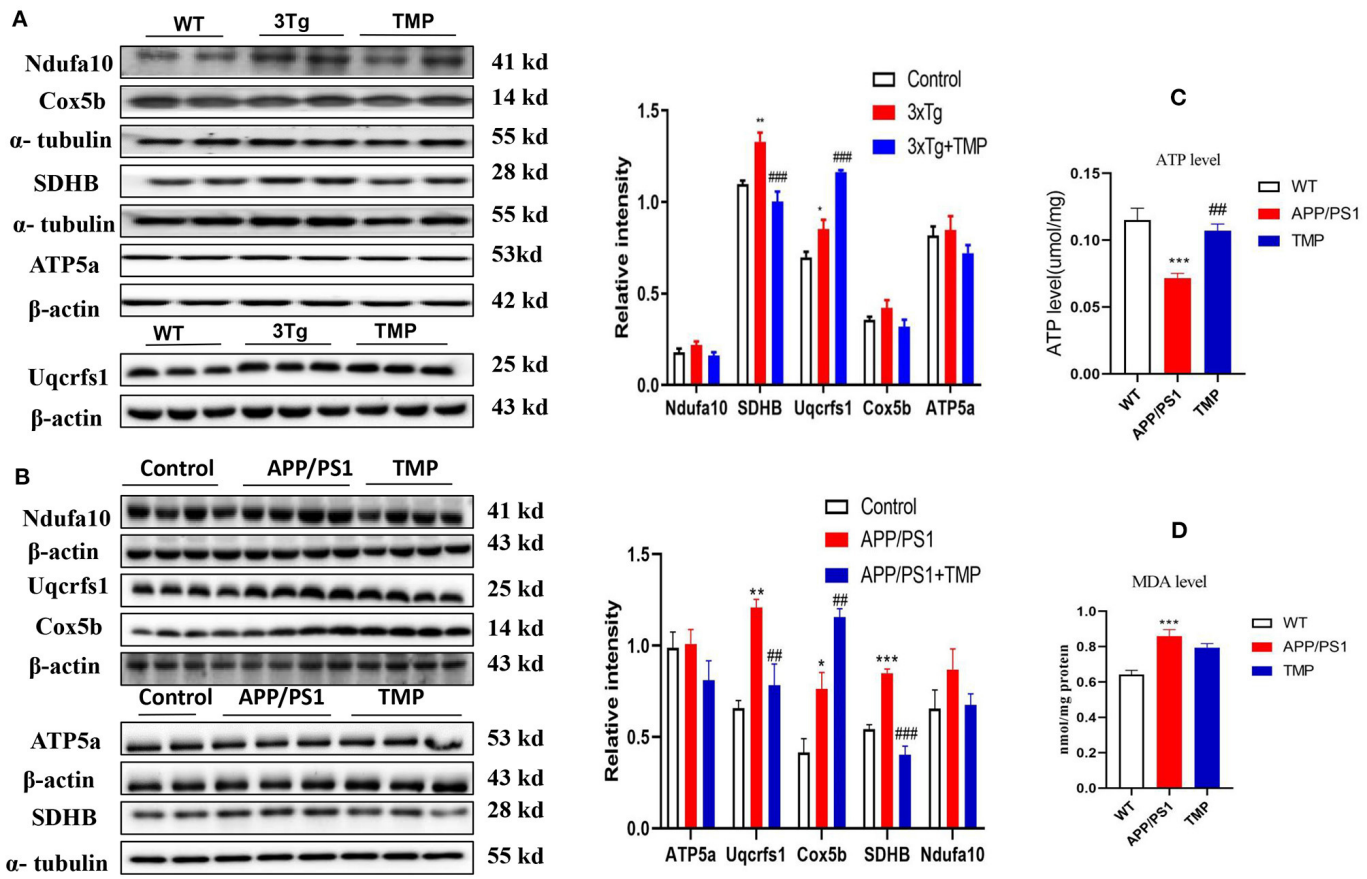
Relative levels of mitochondrial electron transport chain-related brain cortical proteins in 3xTg mice and APP/PS1 mice. For 3xTg-AD mice, the electron transport chain proteins Uqcrfs1, ATP5a, SDHB and Cox5b were measured by Western blot and quantitative analysis **(A)**. For APP/PS1 mice, the electron transport chain proteins Uqcrfs1, ATP5a, SDHB, and Cox5a were measured by Western blot analysis **(B)**. *N* = 4. ATP levels **(C)**, MDA levels **(D)** in cortical tissue of APP/PS1 mice. *N* = 4–5. ^*###*^*p* < 0.001, ^*##*^*p* < 0.01, vs. 3xTg mice, ***p < 0.001, **p < 0.01, *p < 0.05 vs. WT mice; ^*###*^*p* < 0.001, ^*##*^*p* < 0.01 vs. APP/PS1 mice, ***p < 0.001, **p < 0.01, *p < 0.05 vs. WT mice.

Western-blot analysis was performed on the electron transport chain-related proteins, Complex I (NDUFA10), Complex II (SDHB), Complex III (UQCRFS1), Complex IV (COX5B), and Complex V (ATP5A). The level of SDHB in 3xTg-AD mice was significantly modified to the level of WT mice (Figure 10A). In APP/PS1-AD mice, SDHB and UQCRFS1 levels were modified to the level of WT mice (Figure 10B). Compared with WT mice, ATP levels were significantly lower and MDA levels were significantly higher in APP/PS1 mice, and ATP levels were significantly higher and MDA did not change significantly after TMP treatment (Figures 10C,D).

### TMP Inhibited APP Processing and Aβ Accumulation

**Figure 11:**
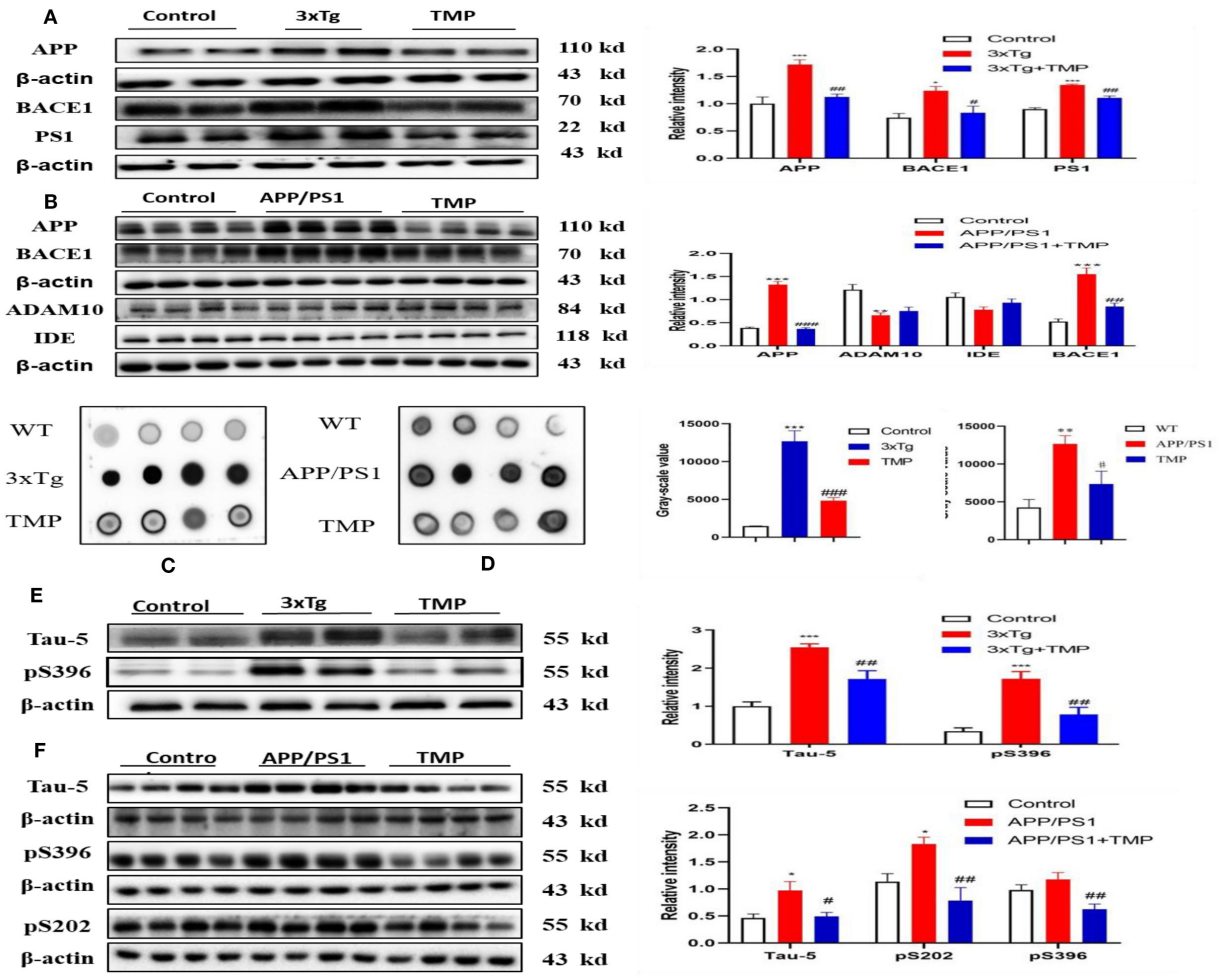
TMP reduced APP levels and p-tau levels in the hippocampus. The proteins related to APP processing by Western blot analysis of 3xTg-AD mice **(A)** and APP/PS1 mice **(B)**. Dot blot analysis was performed to detect Aβ accumulation **(C,D)**. Tau phosphorylation was detected by Western blot analysis **(E,F)**. PS1 and TAU-5 (*N* = 3), others *N* = 4. ^*###*^*p* < 0.001, ^*##*^*p* < 0.01, ^#^*p* < 0.05 vs. 3xTg mice, ***p < 0.001, **p < 0.01, *p < 0.05 vs. WT mice; ^*###*^*p* < 0.001, ^*##*^*p* < 0.01 vs. APP/PS1 mice; ***p < 0.001, **p < 0.01, *p < 0.05 vs. WT mice.

Western-blot analysis showed, that compared with the WT mice, the levels of APP, BACE1, and PS1 in 3xTg mice were significantly increased and the expression of APP, BACE1, and PS1 was significantly decreased after TMP treatment (Figure 11A). Compared with the WT mice, the levels of APP and BACE1 in APP/PS1 mice were significantly increased, while ADAM10 and IDE tended to be decreased, after TMP treatment; the expression of APP, BACE1, and PS1 was significantly reduced, but there was no significant difference in the expression of ADAM10 or IDE (Figure 11B). Dot blot analysis showed that in the cortical tissue of the two models, compared with the WT mice, the level of 6E10 in 3xTg and APP/PS1 mice was significantly increased, while it was significantly decreased after TMP treatment (Figures 11C,D). These results indicated that TMP inhibited APP processing and thus reduced the accumulation of Aβ.

### TMP Reduced p-tau Levels in the Brains of AD Mice

Western-blot analysis showed that the levels of tau-5 and pS396 in 3xTg mice treated with TMP were significantly decreased compared with the untreated 3xTg mice (Figure 11E). Similarly, the levels of tau-5, pS202, and pS396 in APP/PS1 mice treated with TMP were significantly decreased (Figure 11F). These results indicated that TMP could reduce level of p-tau.

## Discussion

The 3xTg and APP/PS1 mouse models have been widely used for AD-related studies (Esquerda-Canals et al., 2017). Among them, 3xTg-AD mice had obvious Aβ deposition in the hippocampus of 3–6 month-old animals, and hyperphosphorylated tau (p-tau) was present by 6–12 months of age (Oddo et al., 2003). APP/PS1-AD mice may exhibit abnormal behavior and Aβ deposition starting from 4 to 6 months of age (Izco et al., 2014). We investigated the therapeutic effect of TMP on cognitive function in the two AD models (3xTg-AD and APP/PS1-AD) and used proteomics methods to explore its potential biological mechanisms of action. Behavioral tests showed that TMP significantly reduced memory impairment of the two AD mouse models. These results are consistent with previous reported studies that TMP can protect against isoflurane-induced cognitive dysfunction in rats (Cui et al., 2020) and restore spatial learning and memory impairment in rats injected with streptozotocin (Lu et al., 2017).

A prominent pathological feature of AD brains is the accumulation of Aβ and NFTs composed of hyperphosphoryled tau (Grontvedt et al., 2018) and a growing body of evidence suggests that that Aβ and tau have synergistic effects in advancing AD-type neurodegeneration (Busche and Hyman, 2020). We found that TMP treatment significantly reduced the levels of Aβ accumulation and tau hyper phosphorylation in the two AD models relative to controls. Beta-secretase 1 (BACE1) plays an important role in APP processing and is closely related to tau protein (Zhang et al., 2018). Our results show that TMP creduces the accumulation of Aβ by inhibiting the activity of BACE1, which could affect levels of p-tau.

We used Venny analysis, GO analysis and protein–protein interaction (PPI) network diagram to analyze the proteomic data and to further explore the molecular mechanisms underlying the apparent murine therapeutic effect of TMP. Venny analysis showed that 116 differentially expressed proteins were commonly changed in 3xTg mice vs. WT mice and TMP-treated mice vs. untreated mice. GO analysis showed that the 116 differential expressed proteins were mainly enriched in processes such as transportation and ion migration, and to participate in MFs such as metal ion binding and ion channel activity. The same 130 differentially expressed proteins were commonly changed in APP/PS1 mice vs. WT mice and TMP-treated mice vs. untreated mice. GO analysis showed that the 130 differential expressed proteins were mainly enriched in processes such as protein transport and protein localization establishment, and to participate in MFs such as protein binding and nucleotide binding. Through proteomics analysis, some proteins functionally related to AD were found in these two mouse models, such as proteins related to mitochondria, GTP binding, synapse, and cytoskeleton. Among them, mitochondria play a significant role in energy production, synaptic transmission, and cognitive function (Picard and McEwen, 2014). Increased reactive oxygen species may impair mitochondrial function may promote neurodegenerative disease (Hroudova et al., 2014). Mitochondrial dysfunction occurred in both the 3xTg and APP/PS1 mice (Coskun et al., 2012).

The differentially expressed proteins located in the mitochondria and mapped the mitochondrial protein profile. Most of the mitochondrial proteins were modified by TMP treatment. For example, FUNDC1 controlled the dynamics and quality of mitochondria by regulating the fission or fusion of mitochondria (Chen M. et al., 2016), while the electron carrier protein CYCS transferred electrons to the cytochrome oxidase complex (Baechler et al., 2019). Western-blot analysis showed that TMP modified the level of mitochondrial Complex II protein SDHB in 3xTg mice and modified the level of mitochondrial Complex II protein SDHB and III protein UQCRFS1 in APP/PS1 mice. APP and Aβ can accumulate on the mitochondrial membrane and interact with mitochondrial components to cause damage to mitochondrial function and structure (Pagani and Eckert, 2011). Mitochondria are central to cellular energy metabolism and the site of most ATP production (Brookes et al., 2004). Mitochondrial dysfunction reduced ATP production in murine AD pathogenesis (Cai and Tammineni, 2017). The increased ATP production after TMP treatment suggested that mitochondrial function was restored in APP/PS1 mice. Aβ, phosphorylated tau protein and the VDAC of the mitochondrial channel interact to cause mitochondrial dysfunction, and synergistic effects of Aβ and tau proteins may cause mitochondrial dysfunction (Manczak and Reddy, 2013). MAPT protein was identified in 3xTg by MS, which was responsible for promoting microtubule assembly and stabilization. WB validation was consistent with proteomic results, with increased tau expression in 3xTg mice compared to WT mice, with decreased expression after TMP administration. APP protein was identified in APP/PS1 mice by MS. APP protein was processed to produce Aβ, which aggregates to form age spots. The WB validation results were consistent with the proteomic results. APP expression was increased in APP/PS1 mice compared to the WT mice and decreased after TMP administration. Aβ and p-tau proteins can directly contribute to mitochondrial dysfunction, and dysfunctional mitochondria in turn accelerate the process of AD (Manczak and Reddy, 2012). It has been reported in the literature that Icariin protects mitochondria and inhibits Aβ production and p-tau protein to improve learning and memory in AD rats (Chen Y. et al., 2016). In our proteomic analysis, mitochondrial proteins were significantly changed after TMP treatment, with improved electron transport chain function and increased ATP levels in the brains of APP/PS1 mice, suggesting that mitochondrial function plays an important role in TMP treatment. The reduction of Aβ and p-tau in the brains of test animals reduced mitochondrial dysfunction, and this was associated with an attenuation of the hallmarks of AD. These data indicated that TMP may protect mitochondrial function in AD mice; this is consistent with reports that systemic TMP treatment restores mitochondrial function and reduces brain damage caused by oxidative stress and cobalt chloride (CoCl2) (Guan et al., 2015). Additionally, TMP can reduce oxidative damage, restore mitochondrial dysfunction and protect HUVEC cells from Hcy-induced apoptosis (Fan et al., 2019).

Synaptic dysfunction also is important to the pathogenesis of AD. Soluble Aβ oligomers reportedly can impair synaptic function (Takahashi et al., 2013), as can tau protein oligomers (Lasagna-Reeves et al., 2011), and Aβ and tau proteins play a synergistic role in synaptic dysfunction (Ittner et al., 2010; Larson et al., 2012). Proteomics showed that TMP modified the expression of some synapse-related proteins. For example, SNAP23 played a major role in transport vesicle docking and fusion, and VAMP2 played a major role in synaptic transmission and neurotransmitter release (Martin et al., 1998; Deak et al., 2004). Consistent with our results in the two mouse models of AD, VAMP2 was down-regulated in AD patients (Pham et al., 2010), but significantly increased after TMP treatment, suggesting that TMP treatment improves synaptic dysfunction.

We noticed that TMP treatment increased some cytoskeleton proteins expression, such as Actin-related protein 2/3 complex subunit 3(Arpc3), Cofilin-1(Cof1), and Arpc3, which is mainly responsible for starting the branches of actin filaments. Mir-29a/B can affect the ARp2/3 complex through the Arpc3 subunit, thus maintaining the flexibility of the neuronal network (Lippi et al., 2011). Cof1 mainly regulates actin cytoskeleton dynamics and plays an important role in neural cell (Gurniak et al., 2005). Our results indicated that TMP may exert a neuroprotective effect by modifying the expression of cytoskeleton proteins.

We found that TMP treatment increased brain ADP-ribosylation factor 6 (ARF6) the two AD model mice. ARF6 is a GTP binding protein, mainly involved in vesicle transport, cytoskeleton, and some other functions (Donaldson, 2003; D'Souza-Schorey and Chavrier, 2006). Activation of ARF6 contributes to the generation of synaptic vesicles in the PC12 nerve (Powelka and Buckley, 2001) and can also recruit AP2/Clathrin-dependent synaptic vesicle membranes to the presynaptic membrane to be endocytosed, thereby accelerating the recovery of neurotransmitters (Krauss et al., 2003). Our results suggest that ARF6 may also be also involved in the neuroprotection of TMP.

## Conclusion

**Figure 12:**
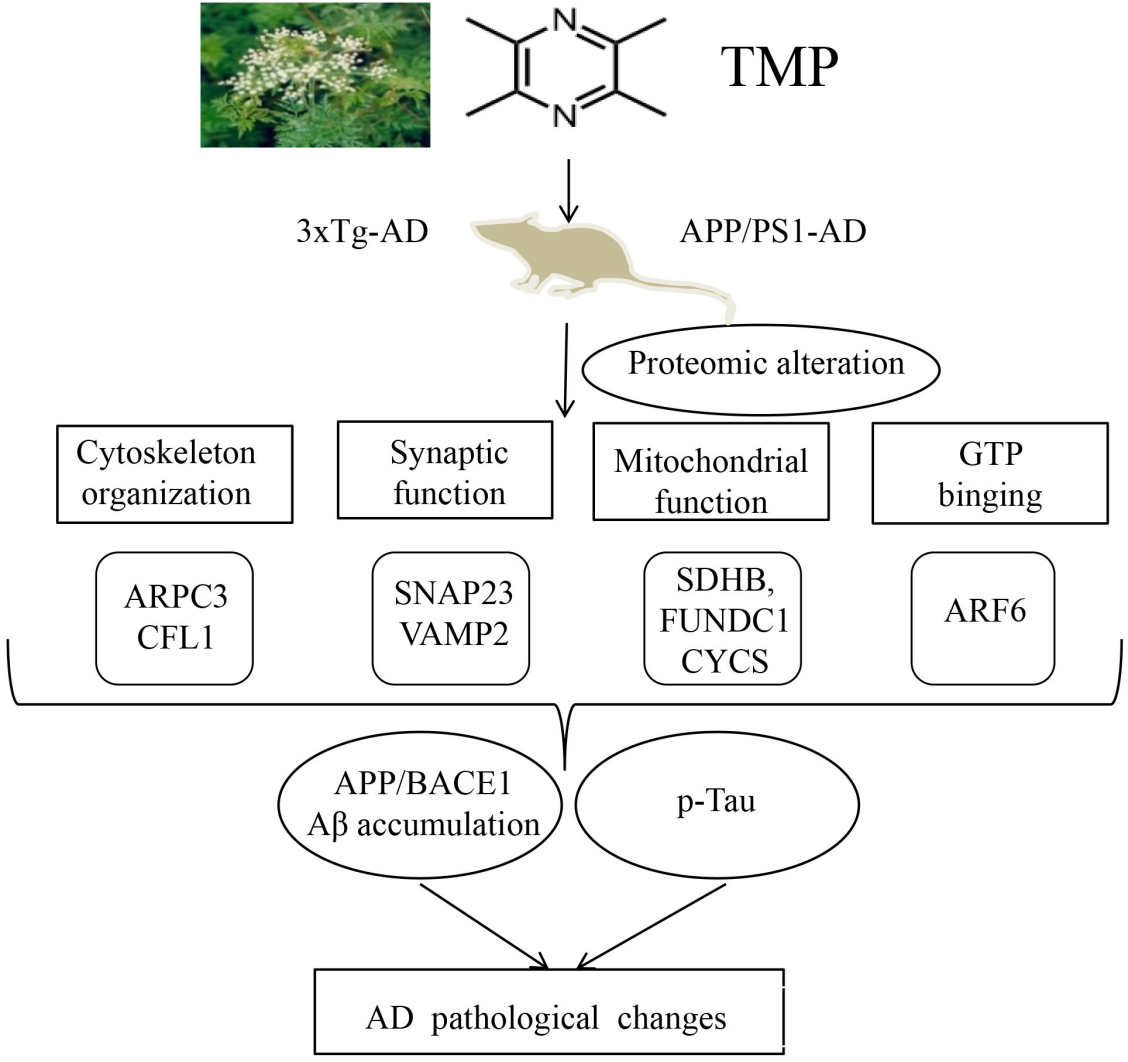
Supposed mode of TMP action. TMP treatment inhibited AD-related changes, including Aβ accumulation and tau phosphorylation, which may be attributed to the modification of mitochondrial function, synaptic function, cytoskeleton, and GTP binding.

In summary, our study of 3xTg-AD and APP/PS1-AD female mice showed that systemic TMP treatment improved memory deficits, reduced Aβ deposition and tau phosphorylation levels, and modified the mitochondrial protein profile, including some oxidative phosphorylation (OXPHOS) proteins. Proteomics suggested that the action of TMP may be closely related to mitochondria, synapses, GTP binding, and cytoskeleton proteins. Although the precise molecular mechanisms remain to be elucidated, our data suggest that TMP has potential for positive therapeutic modification of AD (Figure 12).

## Data Availability Statement

The datasets generated for this study can be found in online repositories. The names of the repository/repositories and accession number(s) can be found below: ProteomeXchange Consortium; PXD022862 and PXD022840.

## Ethics Statement

The animal study was reviewed and approved by All experiments were approved by the Ethics Committee of Shenzhen Center for Disease Control and Prevention.

## Author Contributions

LZ and XY conceived the project, designed the experiments, and wrote the manuscript. XfH and JY designed and performed most of the experiments. XiH performed the informatics analysis and the proteomics analysis. ZZ and JL designed the experiments, analyzed the data, and revised the manuscript. All authors contributed to the article and approved the submitted version.

## Conflict of Interest

The authors declare that the research was conducted in the absence of any commercial or financial relationships that could be construed as a potential conflict of interest.
